# Ultrasound-based radiomics and clinical factors-based nomogram for early intracranial hypertension detection in patients with decompressive craniotomy

**DOI:** 10.3389/fmedt.2025.1485244

**Published:** 2025-02-05

**Authors:** Zunfeng Fu, Lin Peng, Laicai Guo, Chao Qin, Yanhong Yu, Jiajun Zhang, Yan Liu

**Affiliations:** ^1^ Department of Ultrasound, The Second Affiliated Hospital of Shandong First Medical University, Tai'an, China; ^2^ Department of General Practice, The Second Affiliated Hospital of Shandong First Medical University, Tai'an, China; ^3^ Department of Neuro-intensive Care Unit, The Second Affiliated Hospital of Shandong First Medical University, Tai'an, China

**Keywords:** ultrasound imaging, severe traumatic brain injury, intracranial pressure, ultrasound radiomics, machine learning, optic nerve sheath diameter, transcranial color doppler

## Abstract

**Objective:**

This study aims to develop and validate a nomogram that combines traditional ultrasound radiomics features with clinical parameters to assess early intracranial hypertension (IH) following primary decompressive craniectomy (DC) in patients with severe traumatic brain injury (TBI). The study incorporates the Shapley Additive Explanations (SHAP) method to interpret the radiomics model.

**Methods:**

This study included 199 patients with severe TBI (training cohort: *n* = 159; testing cohort: *n* = 40). Postoperative ultrasound images of the optic nerve sheath (ONS) were obtained at 6 and 18 h after DC. Based on invasive intracranial pressure (ICPi) measurements, patients were grouped according to threshold values of 15 mmHg and 20 mmHg. Radiomics features were extracted from ONS images, and feature selection methods were applied to construct predictive models using logistic regression (LR), support vector machine (SVM), random forest (RF), and K-Nearest Neighbors (KNN). Clinical-ultrasound variables were incorporated into the model through univariate and multivariate logistic regression. A combined nomogram was developed by integrating radiomics features with clinical-ultrasound variables, and its diagnostic performance was evaluated using Receiver Operating Characteristic (ROC) curve analysis and decision curve analysis (DCA). The SHAP method was adopted to explain the prediction models.

**Results:**

Among the machine learning models, the LR model demonstrated superior predictive efficiency and robustness at threshold values of 15 mmHg and 20 mmHg. At a threshold of 20 mmHg, the AUC values for the training and testing cohorts were 0.803 and 0.735 for the clinical model, 0.908 and 0.891 for the radiomics model, and 0.918 and 0.902 for the nomogram model, respectively. Similarly, at a threshold of 15 mmHg, the AUC values were consistent across models: 0.803 and 0.735 for the clinical model, 0.908 and 0.891 for the radiomics model, and 0.918 and 0.902 for the nomogram model. Notably, the nomogram model outperformed the clinical model. Decision curve analysis (DCA) further confirmed a higher net benefit for predicting intracranial hypertension across all models.

**Conclusion:**

The nomogram model, which integrates both clinical-semantic and radiomics features, demonstrated strong performance in predicting intracranial hypertension across different threshold values. It shows promise for enhancing non-invasive ICP monitoring and supporting individualized therapeutic strategies.

## Introduction

Monitoring and regulation of intracranial pressure (ICP) are essential in the comprehensive management of various neurological disorders, especially traumatic brain injuries (TBI) (1). Research indicates that up to 72% of TBI patients experience elevated ICP within the first 24 h post-injury (2). Although invasive ICP (ICPi) monitoring is widely used, it is constrained by the need for specialized resources, expertise, and the potential risks it entails, such as infection and hemorrhage (3). These limitations highlight the ongoing need for noninvasive, accurate, and accessible methods of ICP assessment in TBI patients.

It is acknowledged that the role of ICPi monitoring in TBI patients undergoing primary decompressive craniotomy (DC) remains a topic of debate. Proponents argue that maintaining adequate cerebral perfusion pressure (CPP) is essential for TBI management, and that without ICPi monitoring, it is challenging to guide and implement CPP-directed treatments. Studies indicate that many TBI patients continue to experience elevated ICP post-DC, which can result in dangerously low CPP (<60 mmHg), thus making ICPi monitoring critical for informed treatment decisions (4, 5). Conversely, some experts oppose routine ICPi monitoring, suggesting that experienced clinicians can estimate ICP by evaluating pressure at the decompression window and using CT or MRI to detect complications such as recurrent hematomas or changes in ventricular size. They also point to the increased costs and potential risks associated with invasive monitoring. Furthermore, there is no consensus on critical ICP thresholds after primary DC, and for severe TBI, management strategies that focus on maintaining ICP below 20 mmHg have not demonstrated superiority over imaging and clinical examination-based approaches (6, 7). Despite these differing opinions, both sides emphasize the importance of correctly interpreting monitoring data and acknowledge non-invasive ICP monitoring as a valuable alternative.

Among various noninvasive approaches, measuring the optic nerve sheath diameter (ONSD) via ultrasound has emerged as a promising technique due to its simplicity, bedside applicability, and cost-effectiveness (8). The rationale behind this approach is straightforward: elevated ICP causes distension of the optic nerve sheath (ONS), leading to an increase in ONSD. Several studies have shown a strong correlation between ONSD and ICP, establishing it as a valuable parameter for noninvasive ICP assessment (9–11). However, the clinical implementation of ultrasonic ONSD measurement faces challenges due to methodological inconsistencies, including variations in transducer selection, measurement planes, and patient positioning, leading to inaccuracies and limit the generalizability of results (9, 10). Given these limitations, is there an alternative method that could enhance the diagnostic capability for early prediction of increased ICP based on ONSD images? One potential solution is the application of ultrasound radiomics.

Ultrasound radiomics, an emerging and rapidly evolving field, has transformed medical imaging by enabling comprehensive feature extraction and in-depth analysis of tumor phenotypes (12, 13). The confluence of advanced algorithms and artificial intelligence has expanded the scope of radiomic analysis beyond conventional boundaries, particularly in tumor classification and prognosis, as evidenced by a growing body of research that delineates its utility in lesion characterization (13–15). Despite these advancements, there is a paucity of literature quantifying the radiomic features of the ONS on sonographic imaging, an area ripe for exploration in terms of its clinical applicability. In light of this, investigating the comparative efficacy of current machine learning models utilizing ultrasound radiomics for the early diagnosis of intracranial hypertension (IH) is both timely and highly significant.

In this study, we employed different machine learning methodologies to develop and validate an integrated model that combines radiomics features with clinical-ultrasound parameters to predict the onset of early IH under different thresholds (15 mmHg, 20 mmHg) in primary DC patients after TBI. Moreover, the Shapley Additive Explanations (SHAP) technique was utilized to interpret our models. This method helps clarify the significance of individual features within the prediction model and illustrates their contributions to specific predictions (16).

## Materials and methods

### Patient population

**Figure 1:**
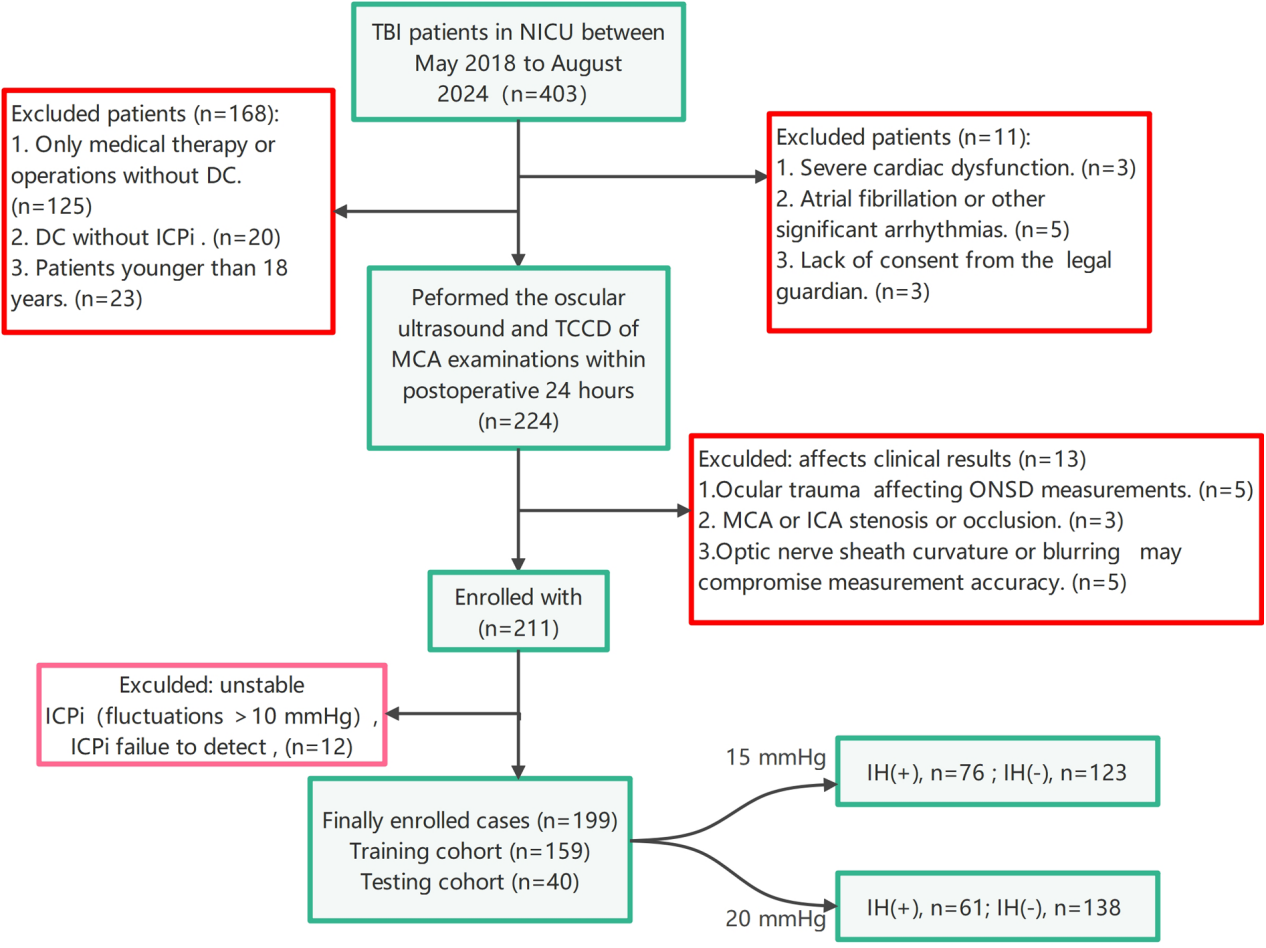
Flowchart for selecting the study population. DC, decompressive craniectomy; ICP, intracranial pressure; ICPi, intracranial pressure invasive; MCA, middle cerebral artery; NICU, neuro-intensive care unit; ONSD, optic nerve sheath diameter; TBI, traumatic brain injury; TCCD, transcranial color doppler; IH, intracranial hypertension.

This retrospective observational study's materials were collected at the Neuro-intensive Care Unit (NICU) of the second hospital affiliated with Shandong First Medical University, located in Taian, Shandong Province, China. It was conducted from May 2018 to August 2024 and was approved by the hospital's Ethics Committee (Nos: 2021-A-037). A total of 199 patients with TBI who underwent primary DC were included after a series of inclusion and exclusion criteria (Figure 1. Flowchart for selecting the study population). All cases had complete two-dimensional ultrasound images of ONS. The cases were randomly divided into a training cohort and a testing cohort in an 8:2 ratio.

### Treatment methods

In TBI patients, the decision to perform primary DC is based on injury mechanism, clinical presentation, Glasgow Coma Scale (GCS) scores, and CT findings. Unilateral DC is performed for hematomas confined to one hemisphere, while bilateral or frontotemporal decompression is used for bilateral hematomas or frontal lobe injuries. The surgical incision typically involves a 9 × 9 cm bone window, extended arcuately to the dura mater. Hematomas, necrotic tissue, and blood clots are removed to ensure hemostasis, and the dura mater is loosely closed or covered with an artificial substitute. ICP is measured using either an intraparenchymal probe or a ventricular catheter connected to an external pressure transducer. The placement of the ICP probe depends on lesion location, ipsilateral for unilateral lesions and on the right side for diffuse injuries. ICP management follows a protocol that includes sedation, optimizing CPP, administering hyperosmolar fluids, and hypothermia, as per institutional guidelines. Given the absence of definitive guidelines for IH following primary DC, we established cutoff values of 15 mmHg (mild elevation) and 20 mmHg (moderate to severe elevation) to assess the model's predictive performance across these thresholds.

### Image acquisition

ONS imagings were performed within 24 h post-DC using the Mindary M9 ultrasound system with a L12-4s probe (1–5 MHz) (Shenzhen, Guangzhou, China). Patients were in a supine position and the probe was applied to the closed upper eyelid with standard gel, angled to capture the ONS in the transverse plane. Adhering to the as low as reasonably achievable (ALARA) principle, the ultrasound output was minimized for safety. Measurements for ONSD were taken 3 mm behind the optic disk, with duplicates in transverse section per eye to reduce variability.

Additionally, bedside middle cerebral artery (MCA) assessments were also recorded by the Mindary M9 ultrasound system equipped with an SP5-1s probe (1–5 MHz). bilateral evaluation of MCA, including pulse index (PI), peak Systolic Velocity (PSV), end-dystolic velocity (EDV), mean velocity (MV). The definitive PI value was ascertained by averaging the two side measurements, or by selecting the higher value in cases of substantial discrepancy.

### Radiological and clinical data analysis

Clinical records and imaging data were obtained from our hospital's routine documentation system and picture archiving and communication system (PACS). A retrospective analysis was conducted on clinical variables, including age, weight, height, body mass index (BMI), gender, ejection fraction (EF), mean arterial pressure (MAP), partial pressures of oxygen (PO2) and carbon dioxide (PCO2) in arterial blood, heart rate, and respiratory rate. Two experienced ultrasound specialists (QC and WQQ), blinded to the ICPi value and clinical data, independently evaluated the ONS images. One specialist had 4 years of experience, and the other had 15 years of expertise in ultrasound examinations. Both had undergone standardized training for ONSD measurements and reached consensus on the image assessments.

### Image segmentation

**Figure 2:**
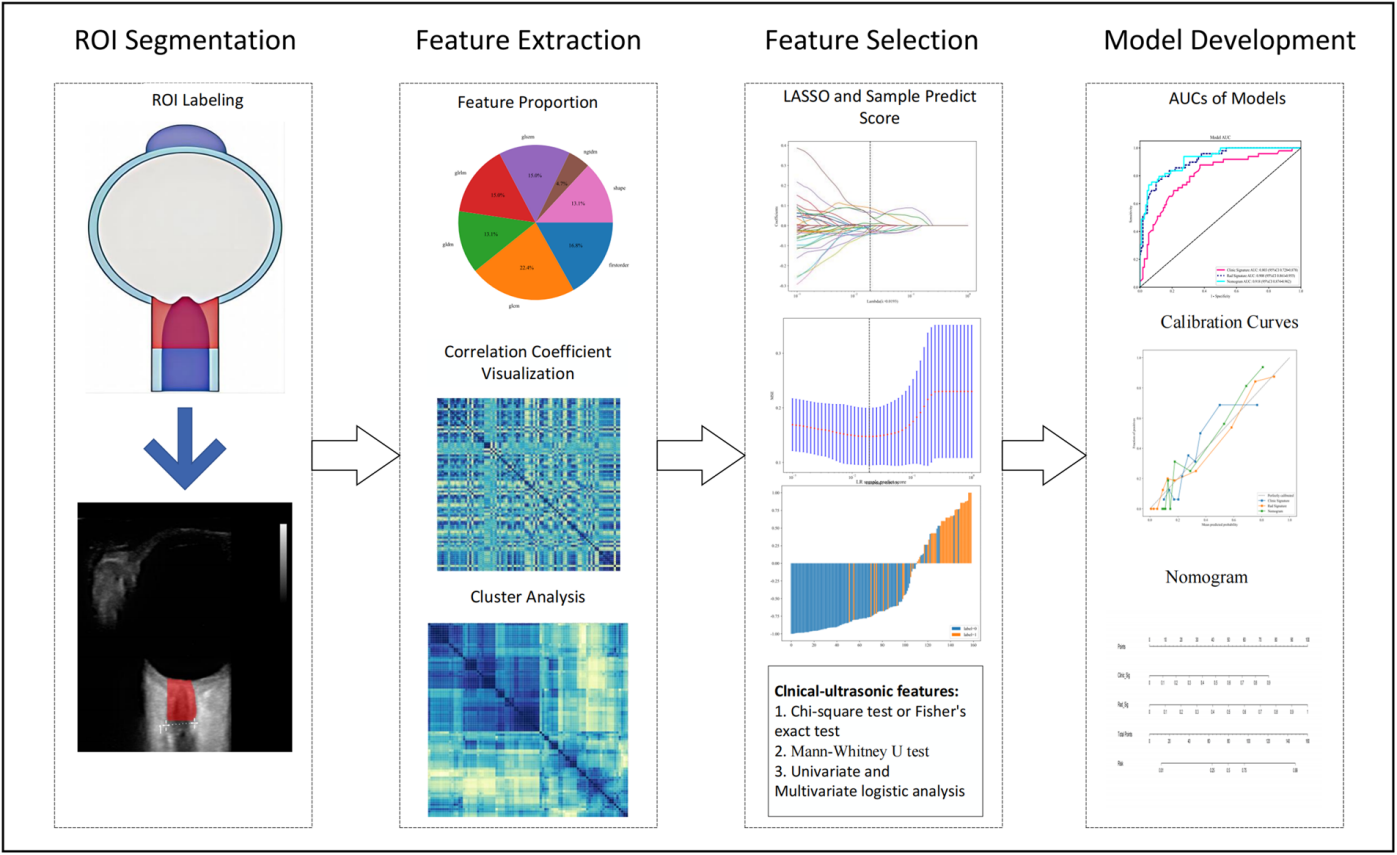
Process of this study. ROI, region of interest.

All US images were stored in Digital Imaging and Communications in Medicine (DICOM) format and were independently reviewed by two experienced ultrasound specialists above. They manually segmented the region of interest (ROI) by using ITK-SNAP software (version3.8.0, http://www.itksnap.org/). A complete schematic diagram is depicted in Figure 2.

The reliability of intra- and inter-observer reproducibility was assessed using the intraclass correlation coefficient (ICC). A random sample of 60 patients was selected, including 18 with IH and 42 with normal intracranial pressure (ICP <20 mmHg), for ROI segmentation. This segmentation was performed by the specialists one month before the study began. An ICC value greater than 0.9 was considered to indicate strong agreement.

### Feature extraction and selection

Radiomic features were extracted from the segmented ROI regions using PyRadiomics (http://pyradiomics.readthedocs.io). The extracted features were classified into three main categories: (I) geometry, (II) intensity, and (III) texture. Geometric features characterize the two-dimensional shape of ONS, while intensity features represent the statistical distribution of voxel intensities within the ONS. These intensity features reflect the first-order statistical distribution of voxel intensities, providing insight into the ONS's overall brightness and contrast. Texture features capture the patterns and spatial distribution of intensities at higher orders, offering additional information on the ONS's internal structure. Specifically, the extraction of textural features employs a variety of methods such as the gray-level co-occurrence matrix (GLCM), gray-level run length matrix (GLRLM), gray level size zone matrix (GLSZM), Gray level dependence matrix (GLDM) and neighborhood gray-tone difference matrix (NGTDM).

For feature Selection, Z-score normalization was applied to standardize all features. Statistical analysis was performed using the Mann–Whitney *U*-test, and features were selected based on their *p*-values, with only those having a *p*-value <0.05 retained. To evaluate feature repeatability, Spearman's rank correlation coefficient was computed. Features with a correlation coefficient greater than 0.9 were considered highly correlated, and one of the correlated features was excluded. The signature construction from the discovery dataset was carried out using Least Absolute Shrinkage and Selection Operator (LASSO) regression. LASSO regression applies the regularization parameter λ to shrink regression coefficients toward zero, eliminating irrelevant features by assigning them a coefficient of zero. The optimal value of λ was determined through 10-fold cross-validation, selecting the value that minimized cross-validation error. LASSO regression modeling was performed using the scikit-learn package in Python.

### Development of machine learning models and nomogram

After LASSO feature selection, the refined set of features was used to develop radiomics models. To identify the classifier with the highest performance in recognizing IH, we evaluated four widely used machine learning algorithms: logistic regression (LR), support vector machine (SVM), random forest (RF), and K-Nearest Neighbors (KNN). The diagnostic efficacy of these models was compared based on metrics such as the area under the receiver operating characteristic curve (AUC), specificity, positive predictive value (PPV), and negative predictive value (NPV). The optimal radiomics model was determined based on these comparisons.

For clinical-ultrasonic variables, univariate logistic regression was first performed to identify significant predictors. A subsequent multivariable analysis refined the final variables in the training group to build the model. Odds ratios (ORs) and 95% confidence intervals (CIs) were calculated for each factor. The same machine learning models used for the radiomic signature were applied, with 5-fold cross-validation and a fixed test cohort for consistent evaluation.

The radiomics nomogram was created by integrating both radiomic and clinical data. Its diagnostic performance was validated in the test cohort through receiver operating characteristic (ROC) analysis. Calibration accuracy was assessed using calibration plots and the Hosmer–Lemeshow test. The clinical applicability of the predictive models was evaluated through decision curve analysis (DCA).

### Statistic analysis

We utilized MedCalc (version 20.015.0) for standard statistical analysis, including Student's *t*-test or Mann-Whitney *U*-test for continuous variables (mean ± SD) and the chi-square or Fisher's exact test for categorical variables (ratios). For the extraction of ultrasound radiomic features, Python (version 3.8.2) with PyRadiomics was employed. The Python scikit-learn package was used for LASSO regression modeling. We considered *P*-values <0.05 as statistically significant.

## Results

### Patients’ population and clinical-ultrasonic characteristics

A total of 199 patients, comprising 130 males and 69 females, were enrolled in our research, with 159 randomly allocated to the training set and 40 to the test set. According to different ICP thresholds, at 20 mmHg, the proportion of hypertension IH was 30.65%, and at 15 mmHg, the proportion of IH was 38.19%. The Reasons for DC, including: Cerebral contusion/lacer-ation (*n* = 34, 17.09%), Acute Intracerebral hematoma (*n* = 58, 29.15%), Acute subdural hematoma (*n* = 107, 53.77%).

No significant disparities were observed between these cohorts in (age, weight, height, BMI, gender, EF, MAP, PO_2_, PCO_2_, heart rate, and respiratory rate), as detailed in Table 1 and Supplementary S1. Notable distinctions (PI, ONSD, MV, EDV) were found in the training set between normal and elevated ICP groups, as well as in the validation set (PI, ONSD). Univariate and multivariable logistic regression analyses also identified PI and ONSD, as significant predictors (Table 2 and Supplementary S2). These variables were integrated into a clinical predictive model. Patients in the elevated ICP group (≥20 mmHg) had a higher likelihood of elevated PI (OR, 4.965; 95% CI, 1.739–18.247), increased ONSD (OR, 3.609; 95% CI, 1.73–7.523), and reduced MV (OR, 0.723; 95% CI, 0.685–0.886), as well as decreased EDV (OR, 0.914; 95% CI, 0.735–0.993). At the 15 mmHg threshold, a similar trend was observed, with elevated PI (OR, 1.047; 95% CI, 1.002–1.073), increased ONSD (OR, 10.912; 95% CI, 4.332–27.716), and reduced MV (OR, 0.955; 95% CI, 0.925–0.987), along with decreased EDV (OR, 0.947; 95% CI, 0.912–0.983).

**Table 1:** Baseline characteristics of patients in cohorts.

Variables	Training cohort (*n* = 159)				Testing cohort (*n* = 40)			
	All (*n* = 159)	Normal (*n* = 110)	Elevated(≥ 20 mmHg) (*n* = 49)	*P* value	All (*n* = 40)	Normal (*n* = 28)	Elevated(≥ 20 mmHg) (*n* = 12)	*P* value
Age (years)	60.52 ± 10.93	59.88 ± 11.05	61.94 ± 10.61	0.354	62.50 ± 9.40	63.07 ± 9.71	61.17 ± 8.90	0.564
Weight (kg)	74.20 ± 10.89	73.95 ± 11.29	74.76 ± 10.03	0.862	75.15 ± 12.10	73.54 ± 12.70	78.92 ± 10.05	0.201
Height (cm)	168.48 ± 7.91	168.19 ± 8.32	169.14 ± 6.91	0.397	168.68 ± 7.25	168.07 ± 7.29	170.08 ± 7.27	0.428
BMI (kg/m^2^)	26.07 ± 2.76	26.08 ± 2.91	26.07 ± 2.42	0.984	26.36 ± 3.44	26.00 ± 3.77	27.22 ± 2.42	0.309
EF (%)	0.63 ± 0.05	0.64 ± 0.06	0.63 ± 0.05	0.584	0.62 ± 0.05	0.62 ± 0.05	0.61 ± 0.06	0.373
PI	1.19 ± 0.42	1.08 ± 0.37	1.43 ± 0.42	<0.001*	1.21 ± 0.39	1.15 ± 0.31	1.34 ± 0.54	0.04*
MAP (mm Hg)	86.79 ± 7.56	86.34 ± 6.92	87.80 ± 8.81	0.476	89.20 ± 9.84	88.96 ± 9.31	89.75 ± 11.40	0.847
ONSD (mm)	4.76 ± 0.50	4.65 ± 0.41	5.00 ± 0.60	<0.001*	4.84 ± 0.50	4.72 ± 0.43	5.13 ± 0.57	0.01*
PaO_2_ (mm Hg)	121.06 ± 24.83	122.07 ± 25.46	118.80 ± 23.44	0.444	119.35 ± 24.23	126.61 ± 24.28	102.42 ± 13.69	0.09
PaCO_2_ (mm Hg)	34.89 ± 2.63	34.93 ± 2.70	34.80 ± 2.50	0.633	35.95 ± 2.41	35.54 ± 2.28	36.92 ± 2.50	0.097
Respiratory rates (bpm)	20.57 ± 3.01	20.48 ± 2.96	20.76 ± 3.14	0.526	20.18 ± 3.10	19.64 ± 2.57	21.42 ± 3.94	0.133
Heart rates (bpm)	84.21 ± 8.22	83.61 ± 7.42	85.57 ± 9.73	0.165	84.20 ± 7.91	83.57 ± 7.67	85.67 ± 8.62	0.45
MV (cm/s)	62.54 ± 22.82	66.35 ± 23.24	53.97 ± 19.47	0.003*	62.04 ± 19.04	61.56 ± 17.31	63.17 ± 23.42	0.745
PSV (cm/s)	107.04 ± 29.30	107.35 ± 27.75	106.34 ± 32.80	0.966	111.17 ± 25.30	110.60 ± 24.88	112.50 ± 27.33	0.831
EDV (cm/s)	37.61 ± 14.69	40.15 ± 14.54	31.92 ± 13.51	<0.001*	39.11 ± 11.94	41.02 ± 11.56	34.67 ± 12.11	0.098
Sex				0.213				1.0
Male	104 (65.41)	68 (61.82)	36 (73.47)		26 (65.00)	18 (64.29)	8 (66.67)	
Female	55 (34.59)	42 (38.18)	13 (26.53)		14 (35.00)	10 (35.71)	4(33.33)	

* Represents *P* < 0.05. Numerical data are presented as mean ± standard deviation. Categorical data as numbers (n%); bpm, breaths per minute or beats per minute; EDV, end-dystolic velocity of MCA; EF, ejection fraction; MAP, mean arterial pressure; MCA, middle cerebral artery; MV, mean velocity of MCA; PCO2, partial pressure of carbon dioxide in arterial blood; PI, pulse index of MCA; PO2, partial pressure of oxygen in arterial blood; PSV, peak systolic velocity of MCA.

**Table 2:** Univariate and multivariable logistic regression analyses for selecting clinical features of model development (ICP ≥ 20 mmHg).

Variable	Univariate analysis		Multivariate analysis	
	OR (95% CI)	*p*-value	OR (95% CI)	*p*-value
Age (years)	1.011 (0.983–1.041)	0.442		
Weight (kg)	1.014 (0.987–1.042)	0.319		
Height (cm)	1.019 (0.98–1.061)	0.330		
BMI (kg/m^2^)	1.028 (0.926–1.142)	0.600		
EF (%)	0.032 (0.001–11.649)	0.261		
PI	6.971 (2.925–16.616)	<0.0001	4.965 (1.739–18.247)	0.011*
MAP (mm Hg)	1.020 (0.983–1.058)	0.293		
ONSD (mm)	4.733 (2.345–9.553)	<0.0001	3.609 (1.732–7.523)	0.004*
PaO_2_ (mm Hg)	0.988 (0.975–1.001)	0.052		
PaCO_2_ (mm Hg)	1.024 (0.913–1.150)	0.685		
Respiratory rates (bpm)	1.064 (0.964–1.175)	0.218		
Heart rates (bpm)	1.031 (0.993–1.07)	0.114		
Sex	0.639 (0.331–1.233)	0.182		
EDV	0.957 (0.933–0.981)	0.0005	0.914 (0.735–0.993)	0.005*
MV	0.978 (0.963–0.994)	0.006	0.723 (0.685–0.886)	0.001*
PSV	0.994 (0.989–1.010)	0.917		

*Represents *p* < 0.05. OR, odds ratio; CI, confidence interval; bpm, breaths per minute or beats per minute; EDV, end-dystolic velocity of MCA; EF, ejection fraction; MAP, mean arterial pressure; MCA, middle cerebral artery; MV, mean velocity of MCA; PCO_2_, partial pressure of carbon dioxide in arterial blood; PI, pulse index; PO_2_, partial pressure of oxygen in arterial blood; PSV, peak systolic velocity of MCA.

### Radiomic signature models and performances

**Figure 3:**
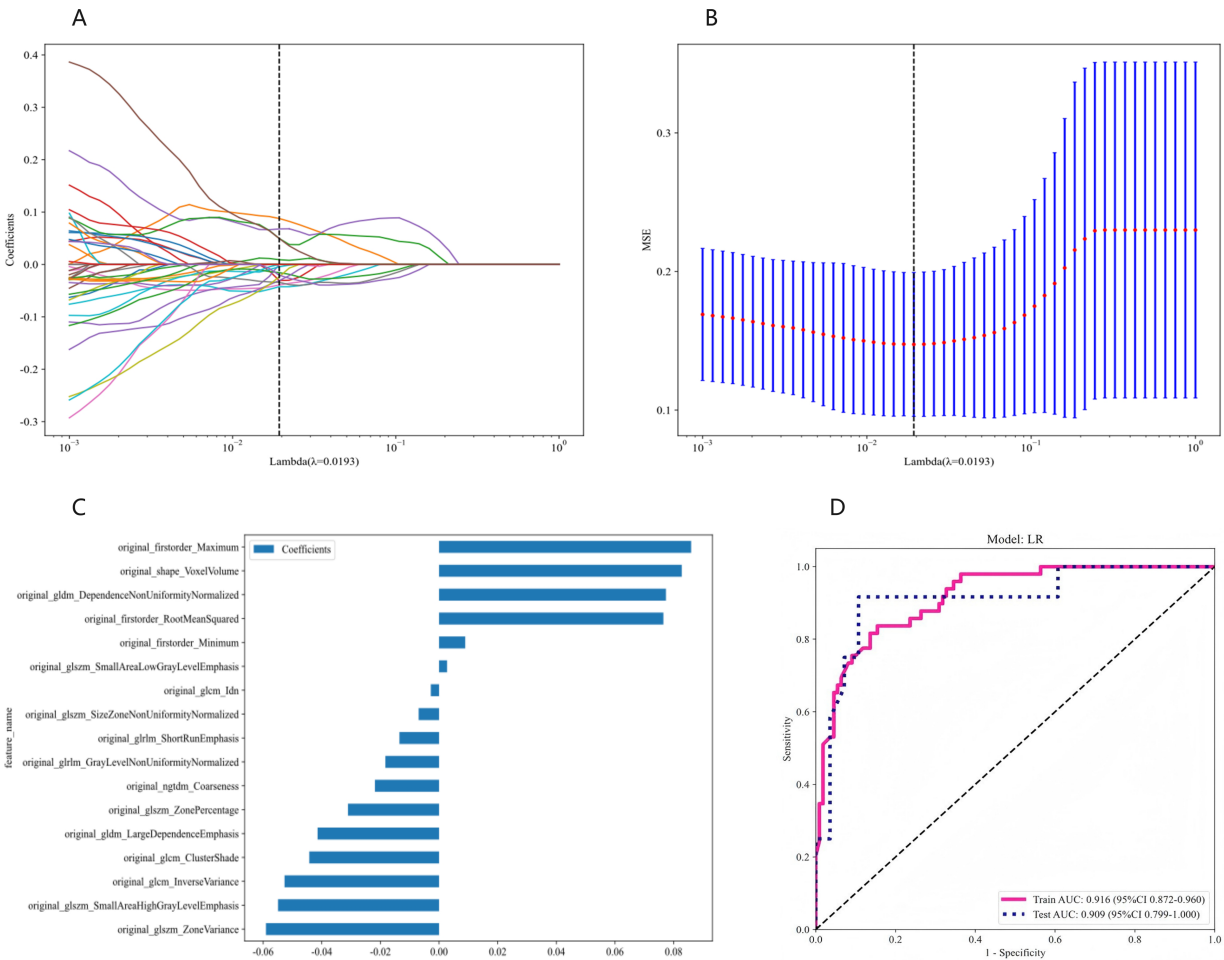
Radiomics feature selection using the LASSO algorithm and performance of the radiomics signature model. **(A)** LASSO coefficient profiles for each feature. Different colored lines represent the corresponding coefficients of each feature. **(B)** Selection of the tuning parameter (*λ*) in the LASSO model. **(C)** Feature weight coefficients after selection. **(D)** ROC curves for the radiomics signature LR model in both the training and testing cohorts. LASSO, least absolute shrinkage and selection operator; ROC, receiver operating characteristic.

A total of 107 handcrafted features are extracted, including 18 first order features, 14 shape features, and the last are texture features. The Mann-Whitney *U*-test identified 87 radiomic features with statistical significance. Following the Spearman rank correlation test, 49 features were selected for further evaluation. The Rad score was calculated using a LASSO regression model, which identified 14 features at 20 mmHg and 18 features at 15 mmHg with nonzero coefficients (depicted in Supplementary Table S3). The coefficients and mean standard errors (MSE) obtained from 10-fold cross-validation are presented in Figures 3A,B, while Figure 3C shows the coefficient values for the final selected features at 20 mmHg. For the 15 mmHg data, refer to Supplementary Figure S1.

**Figure 4:**
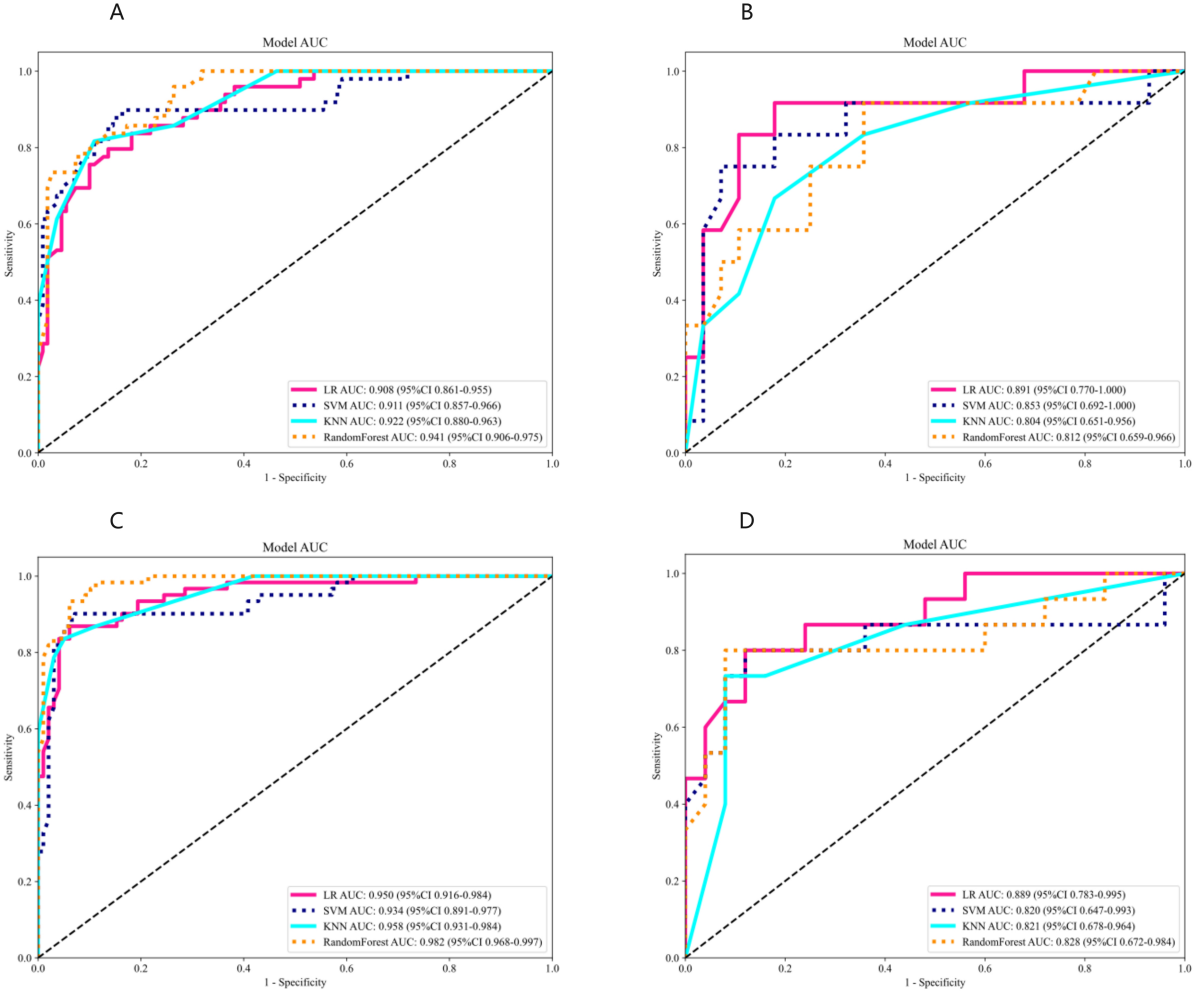
ROC analysis of different models on rad signature. **(A)** ROC curves for the training cohort at 20 mmHg. **(B)** ROC curves for the testing cohort at 20 mmHg. **(C)** ROC curves for the training cohort at 15 mmHg. **(D)** ROC curves for the testing cohort at 15 mmHg. LR, logistic regression; SVM, support vector machine; RF, random forest; AUC, area under the curve; ROC, receiver operating characteristic.

Figure 4 illustrates the ROC curves for four distinct radiomic models: LR, SVM, RF, and KNN, evaluated in both the training and validation cohorts (Figures 4A,B for 20 mmHg, Figures 4C,D for 15 mmHg). In the 20 mmHg training cohort, the RF model demonstrated the highest efficacy, achieving an AUC of 0.941, with an accuracy of 0.893, sensitivity of 0.694, specificity of 0.982, PPV of 0.944, and NPV of 0.878. In contrast, the LR model outperformed the others in the 20 mmHg validation cohort, with an AUC of 0.840, accuracy of 0.853, sensitivity of 0.696, specificity of 0.892, PPV of 0.800, and NPV of 0.858 (see Table 3 for further details). The RF model's performance in both cohorts indicated potential overfitting. Consequently, to ensure robustness and generalizability, the LR model was considered the most suitable for clinical application (Figure 3D). The same situation was also observed in both cohorts at 15 mmHg (Supplementary Figure S1).

**Table 3:** Diagnostic performance of different models for predicting IH in training and test cohorts (≥20 mmHg).

Model_name	Accuracy	AUC	95% CI	Sensitivity	Specificity	PPV	NPV	Task
LR*	0.836	0.908	0.8606–0.9546	0.776	0.864	0.717	0.896	Train
	0.850	0.891	0.7697–1.0000	0.833	0.821	0.667	0.920	Test
SVM	0.849	0.911	0.8566–0.9657	0.878	0.836	0.705	0.939	Train
	0.825	0.853	0.6917–1.0000	0.667	0.929	0.800	0.867	Test
KNN	0.855	0.922	0.8801–0.9630	0.612	0.964	0.882	0.848	Train
	0.750	0.804	0.6512–0.9560	0.417	0.893	0.625	0.781	Test
RandomForest	0.893	0.941	0.9062–0.9751	0.694	0.982	0.944	0.878	Train
	0.700	0.812	0.6590–0.9660	0.833	0.643	0.500	0.900	Test
Clinic signature*	0.667	0.803	0.7276–0.8783	0.898	0.564	0.478	0.925	Train
	0.775	0.735	0.5448–0.9254	0.583	0.857	0.636	0.828	Test
Nomogram*	0.843	0.918	0.8739–0.9621	0.816	0.855	0.714	0.913	Train
	0.925	0.902	0.7637–1.0000	0.833	0.964	0.909	0.931	Test

* Represents models were constructed using LR. LR, logistic regression; SVM, support vector machine; RF, random forest; AUC, area under the curve; PPV, positive prediction value; NPV, negative prediction value.

### Integrated model development and verification

**Figure 5:**
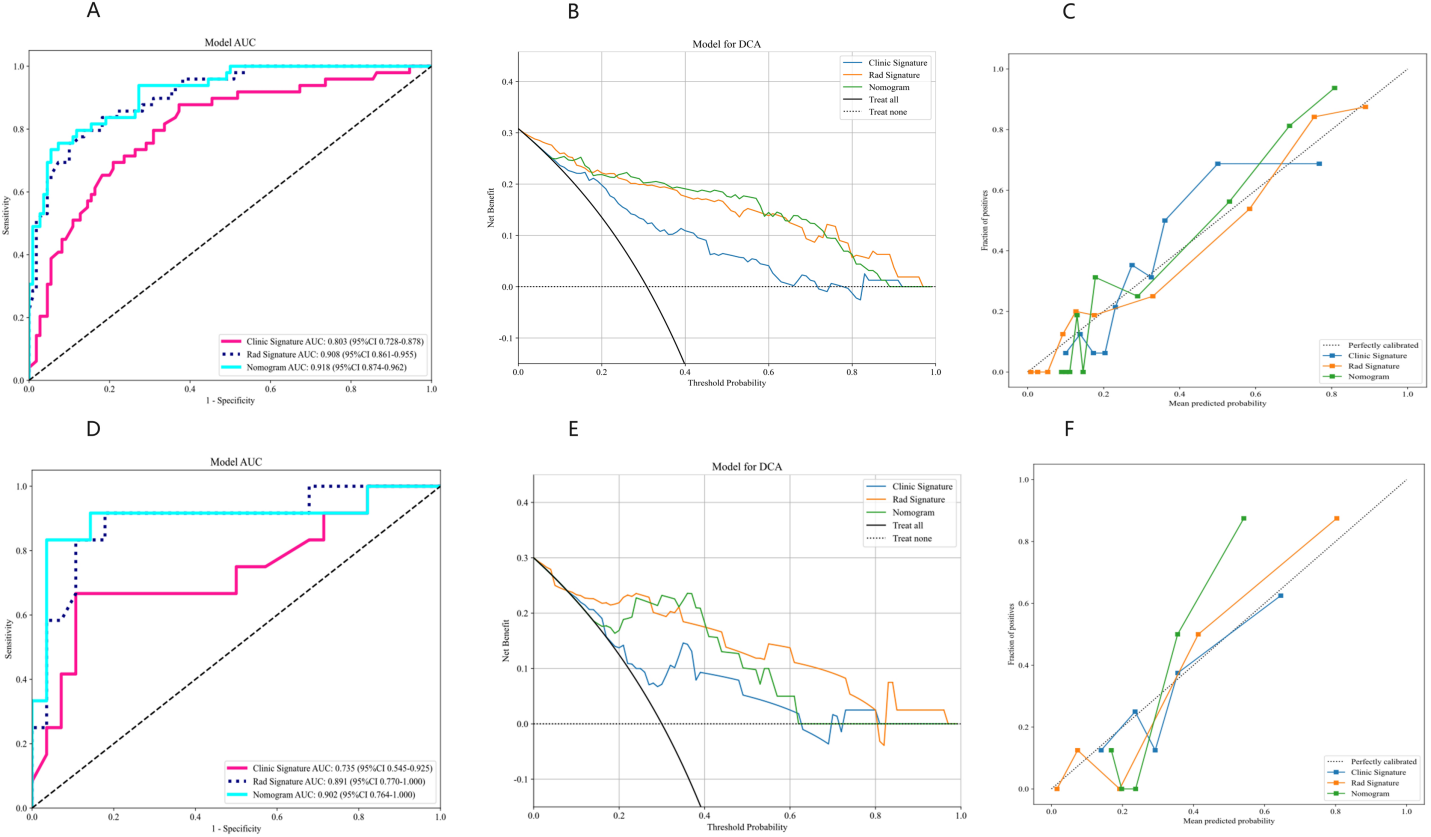
Performance evaluation of clinical, radiomics, and nomogram models using AUC, DCA, and calibration curves. **(A–C)** Results for the training cohort. **(D–F)** Results for the testing cohort. AUC, area under the curve; DCA, decision curve analysis.

**Figure 6:**
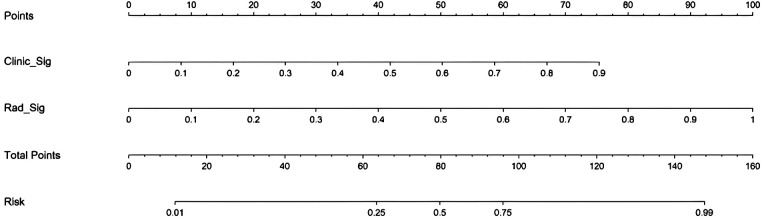
Clinical application of the nomogram in the prediction of IH (≥20 mmHg) in post-DC TBI patients.

At the threshold of 20 mmHg, the performance of the clinical, radiomics, and nomogram models is also summarized in Table 3. In the training cohort (Figure 5A), the clinical model achieved an AUC of 0.803, the radiomics model achieved an AUC of 0.908, and the nomogram model achieved an AUC of 0.918. In the testing cohort (Figure 5D), the clinical model attained an AUC of 0.735, the radiomics model reached an AUC of 0.891, and the nomogram model achieved an AUC of 0.902. The decision curve analysis (DCA) of all three models is shown in Figures 5B,E, demonstrating that, within the training cohort, the nomogram model and radiomics model provided a higher net benefit compared to the clinical model in predicting IH. Calibration curves, presented in Figures 5C,F, illustrate the concordance between the predicted and observed IH in both cohorts. The Hosmer–Lemeshow test indicated no significant difference between the predicted and observed lines in both the training (*P* = 0.319) and testing cohorts (*P* = 0.125). Additionally, the DeLong test confirmed that the nomogram and radiomics models outperformed the clinical model in predictive accuracy in the training cohort (*P* = 0.001), while no significant advantage was observed in the testing cohort (*P* = 0.115). Figure 6 illustrates the nomogram designed for clinical application, where the total score represents the probability of IH (20 mmHg) in post-DC TBI patients. For the 15 mmHg data, refer to Supplementary Figures S2, S3.

### Explanation of prediction models for IH

**Figure 7:**
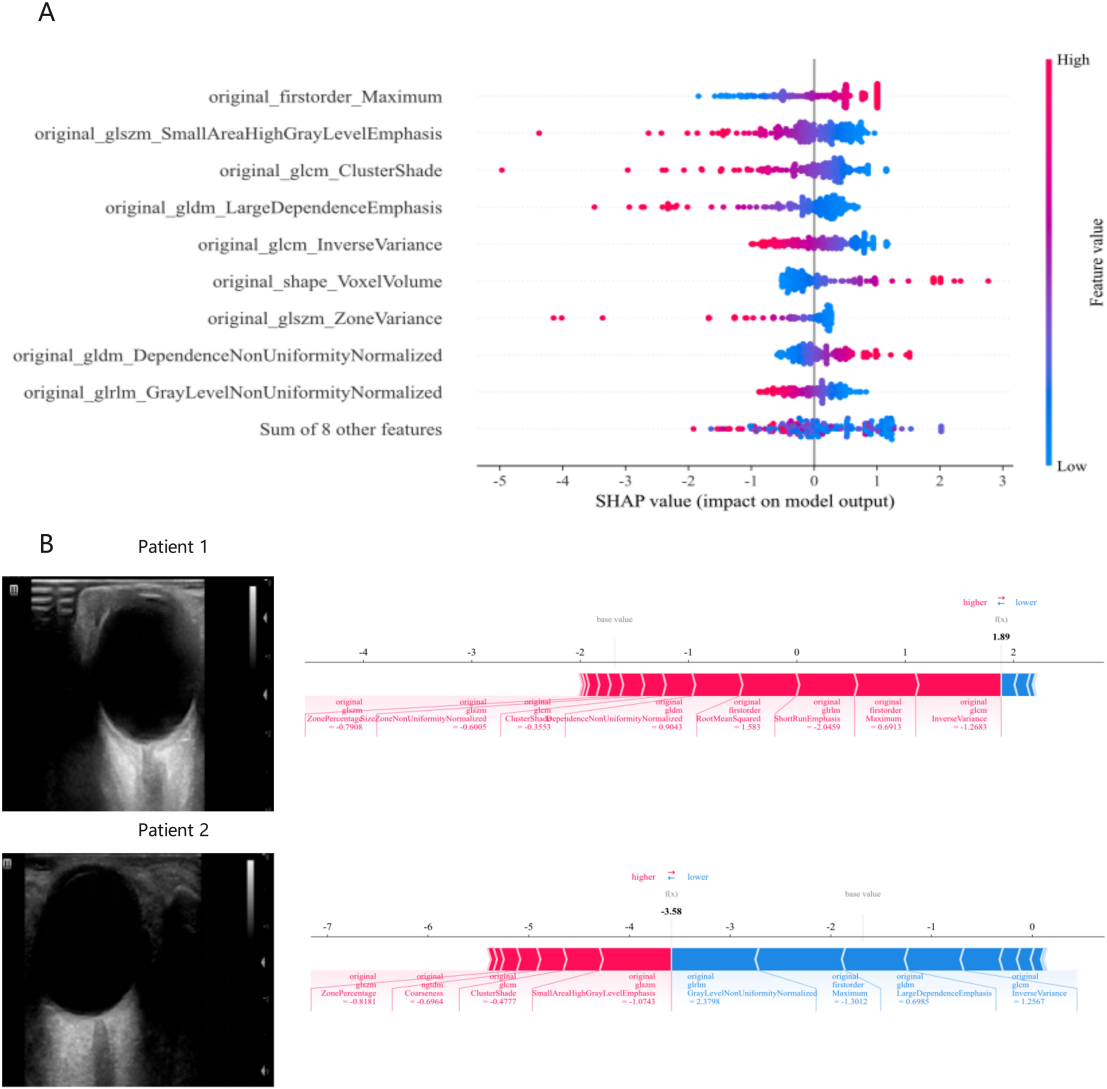
SHAP summary plots for the clinical-radiomic model in predicting intracranial hypertension. **(A)** Feature relevance and the contribution of combined features to the model's predictive performance. **(B)** SHAP force plots illustrating how the clinical-radiomic model distinguishes treatment responses in two patients. Patient 1 has intracranial hypertension, while Patient 2 does not. The data presented in this figure are shown with two decimal places following Z-score normalization. SHAP, Shapley Additive Explanations.

The relationships between features and IH post-DC, which were used to construct the optimal predictive model (clinical-radiomic models), were analyzed using the SHAP algorithm. In the IH prediction model (≥20 mmHg), we identified that features such as original_firstorder_Maximum, original_shape_Voxel Volume, and original_gldm_Dependence NonUniformity Normalized were positively correlated with the occurrence of IH (Figure 7A).

Furthermore, we demonstrated how to interpret the assessment of a single patient within the model. For patient 1, the SHAP value was higher than the baseline value, indicating that this patient exhibited IH. The arrows representing the features highlighted their quantitative contribution to the assessment of elevated intracranial pressure (ICP) (Figure 7B). For instance, the value of the feature *original_glcm_Inverse Variance* was −1.263 for this patient, which positively contributed to the SHAP value. In contrast, for patient 2, who had normal ICP, the SHAP value was noticeably lower than the baseline value. The feature *original_glrm_Gray Level NonUniformity Normalized* had a value of 2.3798, which decreased the SHAP value, indicating a lower likelihood of IH.

## Discussion

Machine learning provides objective, reliable, and accurate models to support clinical decision-making (17). Several studies (18–21) have highlighted its effectiveness in assisting medical practitioners in identifying IH. However, radiomic studies leveraging ONS ultrasound images through machine learning remain limited. Our study demonstrates that ultrasound-based radiomic models not only predict the early onset of increased ICP following DC but also improve diagnostic performance when combined with clinical features in a nomogram. These findings suggest a promising non-invasive assessment method with practical value and reliability for the early detection of IH in post-DC patients.

As is well known, patients undergoing DC do not conform to the Monroe-Kelly doctrine. This is because, after DC, the cranial contents can bulge towards the decompressive bone window. Such changes not only disrupt the original equilibrium state but also affect cerebrovascular autoregulation. Consequently, the traditional pressure-regulating mechanism, which relies on a closed cranial cavity, becomes compromised. In this scenario, the standard invasive ICP thresholds (e.g., 20 mmHg) are no longer applicable to DC patients. Furthermore, the clinical applicability of indices such as the PI, EDV and related non-invasive ICP estimation formulas is also called into question. Although some studies (22, 23) have attempted to assess ICP by observing changes in the ONSD post-DC, these approaches are limited by small sample sizes, poor reproducibility, and high operator dependence, making large-scale clinical application difficult.

Scholars hold differing views on the threshold for defining IH and its management after primary DC (24, 25). Some suggest that when ICP reaches 15 mmHg, brain tissue in post-DC patients may already be suffering from IH-related damage, necessitating proactive clinical interventions. In our study, we utilized two ICP thresholds (15 mmHg and 20 mmHg) to distinguish post-DC IH. The results revealed that radiomic features based on ONS achieved stable AUC values around 0.9 in both the training (0.908 vs. 0.950) and testing cohorts (0.891 vs. 0.889) for both thresholds. In contrast, clinical features performed worse at the 15 mmHg threshold compared to 20 mmHg in both training (0.795 vs. 0.808) and testing (0.681 vs. 0.735) cohorts. This suggests that ONS radiomic features are more advantageous in detecting early-stage ICP elevation.The observed results may be attributed to radiomics extracted from ONS imaging, which uncover subtle imaging features imperceptible to the human eye, providing additional insights for IH prediction. Meanwhile, the limited ultrasonic ONSD expansion from 15 to 20 mmHg, combined with operator measurement errors, contributes to the instability of the clinical model.

Through SHAP analysis, we observed that key radiomic features primarily originate from the GLCM and the GLSZM. GLCM features, such as contrast and correlation, capture the spatial distribution relationships of pixel intensities within the ONS. Early IH leads to ONS enlargement, causing blurred and uneven reflective interfaces within the ROI. These changes alter GLCM feature values, providing critical diagnostic information. GLSZM features, particularly those representing large low-gray regions, highlight changes in the proportion of hypoechoic or anechoic areas within the ONS. These alterations are associated with increased fluid volume and tissue acoustic property changes due to edema, further aiding in the diagnosis of IH. In terms of shape features, parameters such as roundness and aspect ratio are influenced by variations in the shape of the ONS caused by changes in ONSD. Alterations in roundness can effectively quantify these changes, providing valuable diagnostic insights. After rigorous selection and validation, these features collectively characterize the pathophysiological changes of IH from multiple dimensions, offering key information to support model predictions and providing a foundation for clinical interpretation.

Additionally, our clinical data indicate that post-DC patients with IH often exhibit ONS dilation alongside increased PI and reduced EDV and MV, consistent with most studies (26–29). However, minimal variations in respiratory rate, heart rate, and MAP between groups can be attributed to stringent postoperative ICP management, ensuring PaO_2_ and PCO_2_ are maintained within optimal ranges. Although the clinical signature model achieved an AUC of 0.803 in the training set and 0.735 in the test set, these results fall short compared to previous studies (25) reporting AUCs of 0.88–0.99. Discrepancies are largely due to factors such as race, age, and measurement standards significantly influencing ONSD measurements, leading to varying cutoff values across studies (8). Summarizing these findings, we conclude that ONSD and its related clinical models, at the current stage, lack the accuracy and reliability required to replace invasive ICP monitoring, especially in post-DC TBI patients.

Finally, in our comparative analysis of predictive models, LR demonstrated superior predictive performance compared to SVM, RF, and KNN models, consistent with findings reported by Kim (27). This advantage likely stems from the intrinsic linearity of early IH data, which aligns with the linear algorithm of LR, enabling it to outperform the nonlinear approaches of RF and SVM. Although the RF model achieves high accuracy through ensemble learning across multiple decision trees, its generalizability is limited in smaller sample sizes, making it susceptible to overfitting. In contrast, the LR model exhibits greater stability and efficiency, achieving comparable predictive performance with smaller datasets, even when compared to larger training cohorts.

This study has several notable limitations. First, its retrospective and single-center design may have introduced selection bias. Second, there remain ongoing debates regarding the subjective nature of manual segmentation, particularly in establishing boundaries. Future efforts aim to achieve full automation through deep learning techniques. Third, unlike typical TBI cases, post-DC TBI patients do not conform to the Monroe-Kelly doctrine, and their ICP thresholds are less clearly defined. Additional parameters, such as surgical incision size, operation duration, and blood loss, may be necessary for more accurate classification and evaluation. Fourth, the study focused solely on ONSD imaging, overlooking the potential advantages of a multimodal approach, which has shown significant promise in similar contexts. Further prospective studies are needed to explore whether integrating multimodal radiomic data could improve the model's predictive accuracy. Fifth, the small sample size of the training and testing cohorts underscores the need for larger studies to improve the predictive model. Expanding the sample size, using stratified sampling, and applying regularization techniques like LASSO or ridge regression can reduce bias, prevent overfitting, and enhance model stability through further validation.

## Conclusion

Our study introduced and validated an integrated model based on ONS ultrasonic imaging that combines clinical-ultrasonic parameters with radiomics signatures for the early detection of IH within the post-DC TBI patients at different thresholds. The model, optimized with logistic regression (LR), demonstrated superior diagnostic efficacy in our analysis. It offers a novel avenue for precision medicine and has the potential to improve clinical treatment strategies, ultimately aiming to enhance patient outcomes in the critical period following TBI.

## Data Availability

The raw data supporting the conclusions of this article will be made available by the authors, without undue reservation.
