# Supplementary material for: Ultrasound-based radiomics and clinical factors-based nomogram for early intracranial hypertension detection in patients with decompressive craniotomy
Source: Front Med Technol. 2025 Feb 5;7:1485244. doi: 10.3389/fmedt.2025.1485244 (PMC11835818; doi:10.3389/fmedt.2025.1485244)
Supplement: Supplementary file 1 [file Table1.doc]

| Variables  **Table S1. Baseline characteristics of patients in cohorts** | Training cohort (n=159) | | | | Testing cohort (n=40) | | | |
| --- | --- | --- | --- | --- | --- | --- | --- | --- |
|  | All (n=159) | normal (n=98) | elevated(≥ 15 mmHg) (n=61) | P value | All (n=40) | normal (n=25) | elevated(≥ 15 mmHg) (n=15) | P value |
| Age (year) | 60.52±10.93 | 59.80±11.12 | 61.67±10.59 | 0.448 | 62.50±9.40 | 63.44±8.72 | 60.93±10.56 | 0.421 |
| Weight (kg) | 74.20±10.89 | 73.61±11.27 | 75.15±10.27 | 0.497 | 75.15±12.10 | 73.76±13.10 | 77.47±10.22 | 0.354 |
| Height (cm) | 168.48±7.91 | 167.99±8.12 | 169.28±7.55 | 0.183 | 168.68±7.25 | 168.68±6.59 | 168.67±8.48 | 0.995 |
| BMI (kg/m^2^) | 26.07±2.76 | 26.02±2.89 | 26.17±2.56 | 0.731 | 26.36±3.44 | 25.84±3.89 | 27.24±2.37 | 0.217 |
| EF (%) | 0.63±0.05 | 0.63±0.06 | 0.63±0.05 | 0.656 | 0.62±0.05 | 0.62±0.05 | 0.61±0.06 | 0.423 |
| PI | 1.19±0.42 | 1.07±0.36 | 1.37±0.44 | <0.001* | 1.21±0.39 | 1.17±0.33 | 1.28±0.49 | 0.595 |
| MAP (mm Hg) | 86.79±7.56 | 86.37±6.79 | 87.46±8.67 | 0.755 | 89.20±9.84 | 89.92±9.98 | 88.00±9.83 | 0.449 |
| ONSD (mm) | 4.76±0.50 | 4.58±0.34 | 5.03±0.60 | <0.001* | 4.84±0.50 | 4.68±0.43 | 5.11±0.52 | 0.002* |
| PaO_2_ (mm Hg) | 121.06±24.83 | 123.30±25.63 | 117.48±23.25 | 0.151 | 119.35±24.23 | 126.16±24.82 | 108.00±18.94 | 0.019* |
| PaCO_2_ (mm Hg) | 34.89±2.63 | 34.77±2.67 | 35.08±2.58 | 0.650 | 35.95±2.41 | 35.92±2.10 | 36.00±2.93 | 0.920 |
| Respiratory rates (bpm) | 20.57±3.01 | 20.36±2.93 | 20.90±3.13 | 0.238 | 20.18±3.10 | 19.76±2.70 | 20.87±3.68 | 0.345 |
| Heart rates (bpm) | 84.21±8.22 | 83.73±7.67 | 84.98±9.06 | 0.353 | 84.20±7.91 | 83.48±5.91 | 85.40±10.58 | 0.465 |
| MV (cm/s) | 62.54±22.82 | 66.04±22.35 | 56.91±22.62 | 0.011* | 62.04±19.04 | 58.83±16.01 | 67.40±22.83 | 0.234 |
| PSV (cm/s) | 107.04±29.30 | 107.11±26.91 | 106.92±33.00 | 0.907 | 111.17±25.30 | 106.99±24.03 | 118.13±26.65 | 0.181 |
| EDV (cm/s) | 37.61±14.69 | 40.55±14.56 | 32.88±13.74 | 0.001* | 39.11±11.94 | 39.36±10.00 | 38.70±15.01 | 0.624 |
| Sex |  |  |  | 0.373 |  |  |  | 1 |
| Male | 104(65.41) | 61(62.24) | 43(70.49) |  | 26(65.00) | 16(64.00) | 10(66.67) |  |
| Female | 55(34.59) | 37(37.76) | 18(29.51) |  | 14(35.00) | 9(36.00) | 5(33.33) |  |

### *Represents P<0.05. Numerical data are presented as mean ± standard deviation. Categorical data as numbers (n%); bpm breaths per minute or beats per minute, EDV end-dystolic velocity of MCA, EF Ejection Fraction, MAP mean arterial pressure, MCA middle cerebral artery, MV mean velocity of MCA, PCO2 partial pressure of carbon dioxide in arterial blood, PI pulse index of MCA, PO2 partial pressure of oxygen in arterial blood, PSV peak Systolic Velocity of MCA.
