# Supplementary material for: Ultrasound-based radiomics and clinical factors-based nomogram for early intracranial hypertension detection in patients with decompressive craniotomy
Source: Front Med Technol. 2025 Feb 5;7:1485244. doi: 10.3389/fmedt.2025.1485244 (PMC11835818; doi:10.3389/fmedt.2025.1485244)
Supplement: Supplementary file 2 [file Table2.doc]

**Table 2. Univariate and multivariable logistic regression analyses for selecting clinical features of model development (ICP≥15 mmHg)**

| **Variable** | **Univariate analysis** | | **Multivariate analysis** | |
| --- | --- | --- | --- | --- |
|  | **OR (95% CI)** | **p-value** | **OR (95% CI)** | **p-value** |
| Age (years) | 1.021 (0.993-1.047) | 0.462 |  |  |
| Weight (kg) | 1.012(0.985-1.039) | 0.345 |  |  |
| Height (cm) | 1.157(0.88-1.661) | 0.330 |  |  |
| BMI (kg/m^2^) | 1.118 (0.916-1.140 ) | 0.610 |  |  |
| EF (%) | 0.072 (0.001-14.349) | 0.245 |  |  |
| PI | 2.7711 (2.025-11.730) | 0.001 | 1.047(1.002-1.073) | 0.002 |
| MAP (mm Hg) | 1.029 (0.998-1.089) | 0.311 |  |  |
| ONSD (mm) | 5.234(2.125-11.503) | <0.0001 | 10.912 (4.332-27.716) | <0.0001 |
| PaO_2_ (mm Hg) | 0.898 (0.823-1.010) | 0.062 |  |  |
| PaCO_2_ (mm Hg) | 1.124 (0.903-1.111) | 0.697 |  |  |
| Respiratory rates (bpm) | 1.055 (0.974-1.165) | 0.210 |  |  |
| Heart rates (bpm) | 1.001 (0.873-1.112) | 0.127 |  |  |
| Sex | 0.659 (0.531-1.131) | 0.113 |  |  |
| EDV | 0.929(0.913-0.977) | 0.0005 | 0.947(0.912-0.983) | 0.012 |
| MV | 0.918(0.899-0.941) | 0.006 | 0.955(0.925-0.987) | 0.020 |
| PSV | 0.654(0.455-0.992) | 0.622 |  |  |

*Represents p < 0.05. OR odds ratio, CI confidence interval, bpm breaths per minute or beats per minute, EDV end-dystolic velocity of MCA, EF Ejection Fraction, MAP mean arterial pressure, MCA middle cerebral artery, MV mean velocity of MCA, PCO_2_ partial pressure of carbon dioxide in arterial blood, PI pulse index, PO_2_ partial pressure of oxygen in arterial blood, PSV peak Systolic Velocity of MCA.
