# Supplementary material for: Ultrasound-based radiomics and clinical factors-based nomogram for early intracranial hypertension detection in patients with decompressive craniotomy
Source: Front Med Technol. 2025 Feb 5;7:1485244. doi: 10.3389/fmedt.2025.1485244 (PMC11835818; doi:10.3389/fmedt.2025.1485244)
Supplement: Supplementary file 3 [file Table3.doc]

Table S3. Radiomics Features Selected by LASSO Regression.

| Feature category | Feature name | Description |
| --- | --- | --- |
| First order features | original_firstorder_Maximum | The maximum gray level intensity within the ROI. |
|  | original_firstorder_Minimum | The minimum gray level intensity within the ROI. |
|  | original_firstorder_Root Mean Squared | Grayscale fluctuation and contrast indicator. |
|  |  |  |
| Shape features(2D) | original_shape_Sphericity | Quantification of the roundness or sphericity of the ROI |
|  | original_shape_Voxel Volume | Voxel-based volume estimation of ROI |
|  | original_shape_Minor Axis Length | Emphasizes the distribution of shorter runs with lower gray-level intensities in the image. |
|  | original_shape_Maximum3DDiameter | The shortest diameter of the ellipse that has the same normalized second central moments as the 3D shape. |
| Texture features | original_glcm_Cluster Shade | Measure of the skewness and uniformity of the GLCM. |
|  | original_glcm_Inverse Variance | Measure of Texture homogeneity of the GLCM. |
|  | original_gldm_Small Dependence High Gray Level Emphasis | Emphasizes small dependent regions with high gray-level intensities. |
|  | original_gldm_Large Dependence High Gray Level Emphasis | Emphasizes large dependent regions with high gray-level intensities. |
|  | original_gldm_Dependence Non-Uniformity Normalized | Normalized measure of non-uniform gray-level distribution dependence |
|  | original_gldm_Dependence Variance | Variance of gray-level dependence |
|  | original_glrlm_Long Run Low Gray Level Emphasis | Emphasizes long runs with low gray-level intensities. |
|  | original_glrlm_Run Variance | Measures the variance in run lengths, reflecting the heterogeneity of run sizes. |
|  | original_glrlm_Short Run Low Gray Level Emphasis | Emphasizes the distribution of shorter runs with lower gray-level intensities in the image. |
|  | original_glrlm_Gray Level Non-Uniformity Normalized | Normalized measure of gray-level non-uniformity |
|  | original_glszm_Size Zone Non-Uniformity Normalized | Normalized assessment of zone size non-uniformity |
|  | original_glszm_Small Area High Gray Level Emphasis | Emphasis of high gray levels in small areas in the ROI |
|  | original_glszm_Zone Percentage | Percentage of zones within the ROI |
|  | original_glszm_Zone Variance | Dispersion of the sizes of uniform texture zones |

The Formula of Rad Score:

20mmHg: label = 0.3065326633165829 + +0.087757 * original_firstorder_Maximum +0.009198 * original_firstorder_Minimum +0.067706 * original_firstorder_RootMeanSquared -0.039173 * original_glcm_ClusterShade -0.042495 * original_glcm_InverseVariance +0.049721 * original_gldm_DependenceNonUniformityNormalized -0.030653 * original_gldm_DependenceVariance -0.004456 * original_glrlm_GrayLevelNonUniformityNormalized -0.015547 * original_glszm_SizeZoneNonUniformityNormalized -0.030732 * original_glszm_SmallAreaHighGrayLevelEmphasis -0.034103 * original_glszm_ZonePercentage -0.023573 * original_glszm_ZoneVariance -0.020528 * original_shape_Sphericity +0.049583 * original_shape_VoxelVolume

15mmHg: label = 0.38190954773869346 + +0.078755 * original_firstorder_Maximum +0.053337 * original_firstorder_Minimum +0.074400 * original_firstorder_RootMeanSquared -0.048239 * original_glcm_ClusterShade -0.024814 * original_glcm_InverseVariance +0.029406 * original_gldm_DependenceNonUniformityNormalized +0.025961 * original_gldm_LargeDependenceHighGrayLevelEmphasis -0.004831 * original_gldm_SmallDependenceLowGrayLevelEmphasis -0.037495 * original_glrlm_GrayLevelNonUniformityNormalized -0.007001 * original_glrlm_LongRunLowGrayLevelEmphasis -0.006378 * original_glrlm_RunVariance -0.017323 * original_glrlm_ShortRunLowGrayLevelEmphasis -0.022072 * original_glszm_SizeZoneNonUniformity -0.048149 * original_glszm_SmallAreaHighGrayLevelEmphasis -0.059317 * original_glszm_ZonePercentage +0.041194 * original_shape_Maximum3DDiameter +0.022110 * original_shape_MinorAxisLength +0.027520 * original_shape_VoxelVolume
