# Supplementary figures and images for: Ultrasound-based radiomics and clinical factors-based nomogram for early intracranial hypertension detection in patients with decompressive craniotomy
Source: Front Med Technol. 2025 Feb 5;7:1485244. doi: 10.3389/fmedt.2025.1485244 (PMC11835818; doi:10.3389/fmedt.2025.1485244)

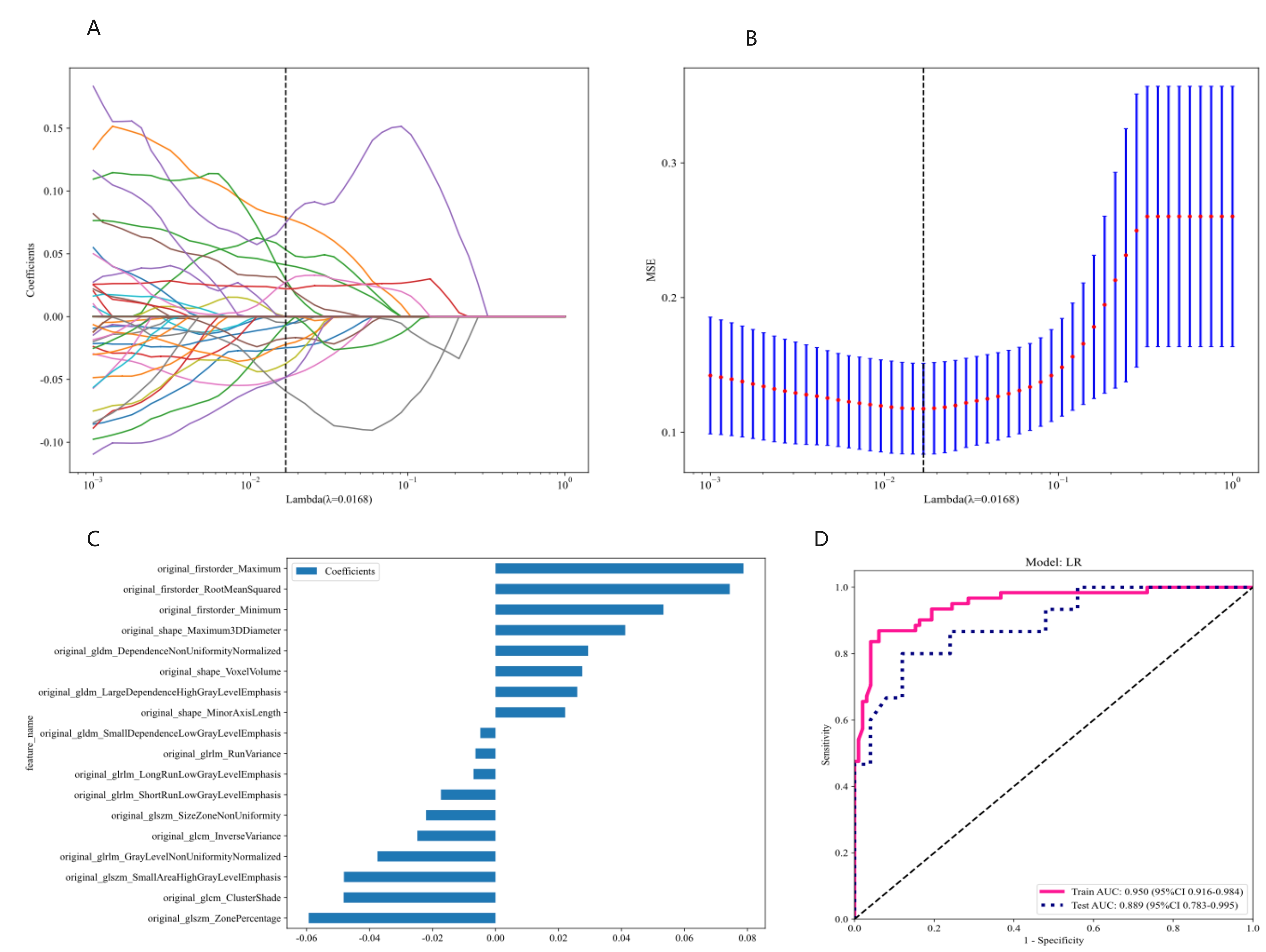

Supplement: Supplementary file 4 [file Image1.jpeg]

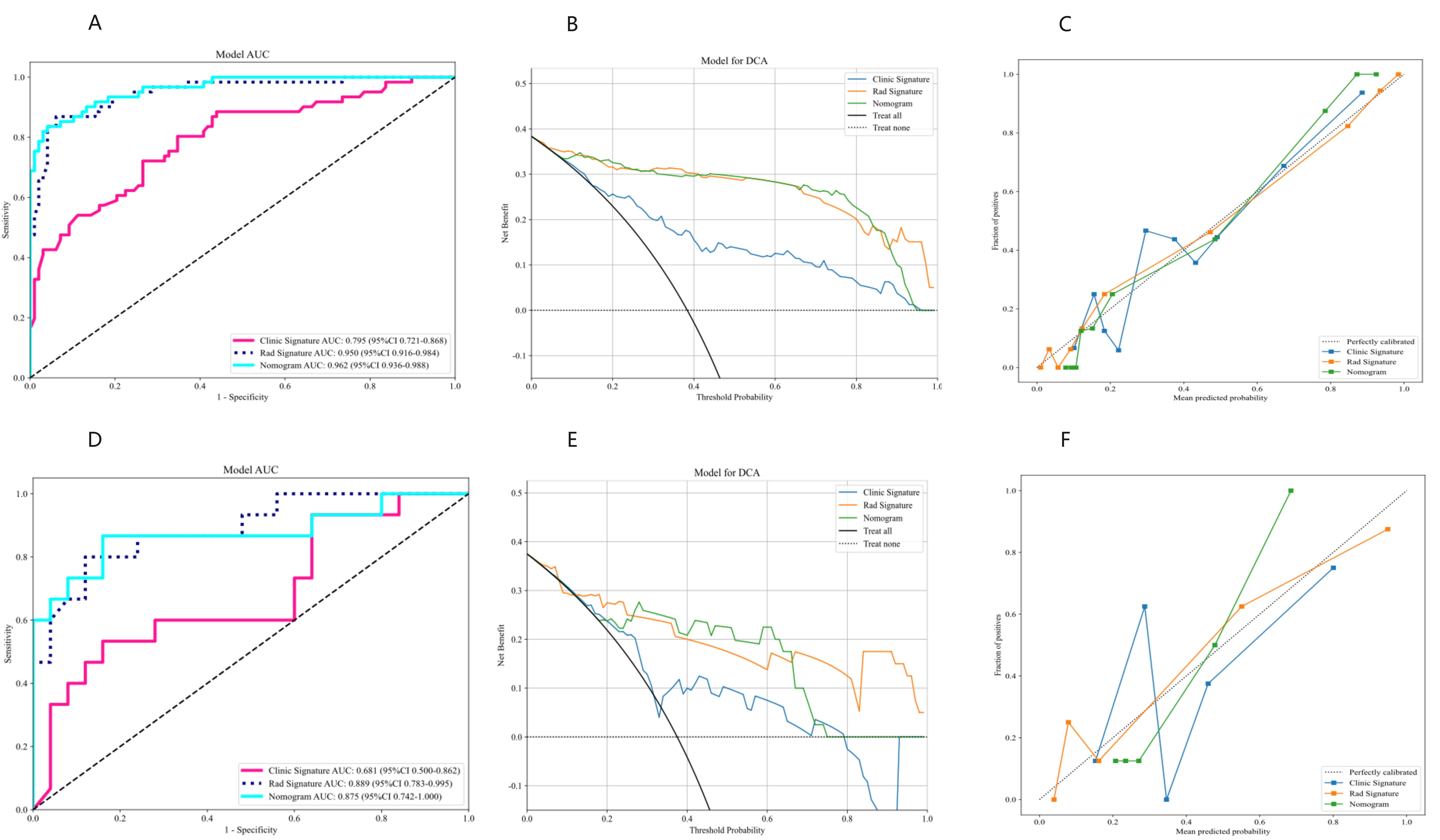

Supplement: Supplementary file 5 [file Image2.jpeg]

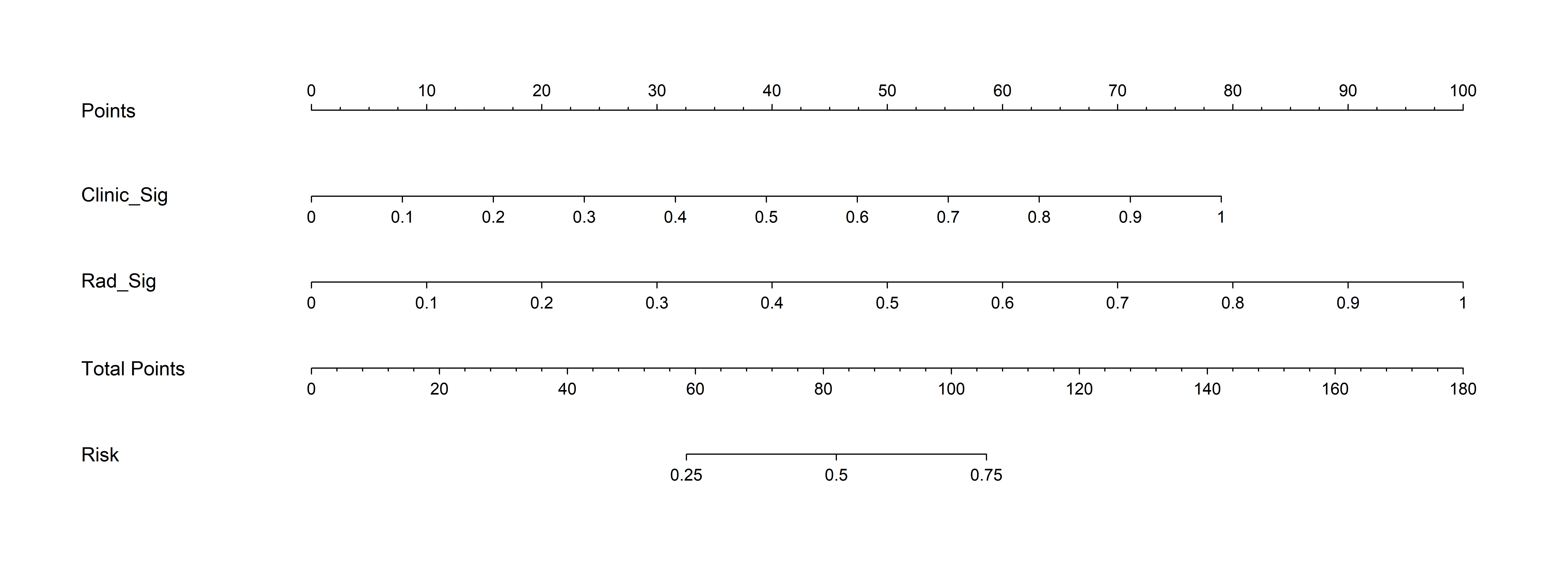

Supplement: Supplementary file 6 [file Image3.jpeg]
